# Examining Mental Workload Relating to Digital Health Technologies in Health Care: Systematic Review

**DOI:** 10.2196/40946

**Published:** 2022-10-28

**Authors:** Lisanne Kremer, Myriam Lipprandt, Rainer Röhrig, Bernhard Breil

**Affiliations:** 1 Faculty of Health Care Niederrhein University of Applied Sciences Krefeld Germany; 2 University Hospital Rheinisch-Westfälische Technische Hochschule Aachen Institute of Medical Informatics Aachen Germany

**Keywords:** mental workload, mental workload measurement, assessment, health care professional, health information system(s), digital health technology, systematic review

## Abstract

**Background:**

The workload in health care is increasing and hence, mental health issues are on the rise among health care professionals (HCPs). The digitization of patient care could be related to the increase in stress levels. It remains unclear whether the health information system or systems and digital health technologies (DHTs) being used in health care relieve the professionals or whether they represent a further burden. The mental construct that best describes this burden of technologies is mental workload (MWL). The measurement methods of MWL are particularly relevant in this sensitive setting.

**Objective:**

This review aimed to address 2 different but related objectives: identifying the factors that contribute to the MWL of HCPs when using DHT and examining and exploring the applied assessments for the measurement of MWL with a special focus on eye tracking.

**Methods:**

Following the PRISMA (Preferred Reporting Items for Systematic Reviews and Meta-Analyses) 2020 statement, we conducted a systematic review and processed a literature search in the following databases: MEDLINE (PubMed), Web of Science, Academic Search Premier and CINAHL (EBSCO), and PsycINFO. Studies were eligible if they assessed the MWL of HCPs related to DHT. The review was conducted as per the following steps: literature search, article selection, data extraction, quality assessment (using the Standard Quality Assessment Criteria for Evaluation Primary Research Papers From a Variety of Fields [QualSyst]), data analysis, and data synthesis (narrative and tabular). The process was performed by 2 reviewers (in cases of disagreement, a third reviewer was involved).

**Results:**

The literature search process resulted in 25 studies that fit the inclusion criteria and examined the MWL of health care workers resulting from the use of DHT in health care settings. Most studies had sample sizes of 10-50 participants, were conducted in the laboratory, and had quasi-experimental or cross-sectional designs. The main results can be grouped into two categories: assessment methods and factors related to DHT that contribute to MWL. Most studies applied subjective methods for the assessment of MWL. Eye tracking did not play a major role in the selected studies. The factors contributing to a higher MWL were clustered into organizational and systemic factors.

**Conclusions:**

Our review of 25 papers shows a diverse assessment approach toward the MWL of HCPs related to DHT as well as 2 groups of relevant contributing factors to MWL. Our results are limited in terms of interpretability and causality due to methodological weaknesses of the included studies and may be limited by some shortcomings in the search process. Future research should concentrate on adequate assessments of the MWL of HCPs dependent on the setting, the evaluation of quality criteria, and further assessment of the contributing factors to MWL.

**Trial Registration:**

PROSPERO (International Prospective Register of Systematic Reviews) CRD42021233271; https://www.crd.york.ac.uk/PROSPERO/display_record.php?ID=CRD42021233271

## Introduction

### Background

The decrease in nursing staff with the simultaneous increase in patients with multiple morbidities in need of care means an increase in workload of the remaining nursing staff. The digitization of health care in theory should help to counteract this change and its consequences. However, in Germany in particular, the process is proceeding very slowly; Germany is ranked 16th out of 17 countries in the Bertelsmann Digital Health Index [1]. The application of digital health technology (DHT) is an important factor in the digitalization process. DHTs in the context of this review means technologies that are directly linked to outpatient and inpatient care and are implemented by nurses or physicians. By DHT, we mean, for instance, health information systems (HISs), medical devices, and other digital applications that support patient care from the perspective of health care professionals (HCPs).

In addition to the positive effects of the use of DHT, there is also evidence which suggests that its use can cause extra workload [2] and can consequently have a negative impact on HCPs’ health [3]. However, it remains ambiguous which factors are specifically responsible for a high mental workload (MWL) during the use of DHT. Initial results show that this may be because of a lack of usability and user involvement as well as poor implementation processes [4,5].

Poor usability and other factors rooted in technology can cause a high MWL [5]. High workloads can cause errors independent of the operators status (novice or expert). Those errors often results form decision-making processes. [6]. When working with patients, however, susceptibility to errors as well as indecisiveness cannot be an option. Working in outpatient and inpatient care can be considered as working in safety-critical environments. Many tasks, varying in complexity, occur within limited time windows. Decisions could be supported by different DHTs through the structured and standardized presentation of information.

The interaction between the users and the systems is complex and interdependent, which contributes to difficulties in the prediction of effects related to the systems on the users [7].

Wickens et al [8] give a good practical example for this effect. During surgery, different complex tasks have to be performed by the surgeon in addition to observing the patient. In the event of a sudden change in the patient's vital signs, which can be potentially life-threatening, the surgeon has to promptly take an appropriate decision on how to proceed. Complex demands could result in an overload if they exceed the capacity of attentional resources [7]. Consequences of overload are an increasing vulnerability to errors and decreasing performance. In addition to serious consequences for patients, an overload also has drastic effects on employees. High workloads caused by several factors (including technology) result in consequences regarding the workers’ health; technostress, mental health issues such as depression or burnout, and decreased job satisfaction are only a few of the alarming effects [9]. There is growing evidence that DHTs are contributing to increasing mental health problems, (eg, burnout of health care workers [10,11]). The investigation of MWL in different situations is a possible approach toward identifying the main causes behind, for example, emerging incidences of burnout in physicians and nurses [12].

### Mental Workload

MWL can be defined using different approaches and is usually influenced by different and multiple factors. It is multidimensional and multifaceted and is one of the most important variables for understanding and predicting human performance.

The possible definitional approaches of workload can be derived from two different perspectives:

1. MWL as an external variable referring to task requirements: the amount of work and the number of tasks to be completed (in a limited time), that is,
  task load
2. Interaction between task and human resources resulting in a subjective psychological experience [13,14]

Eggemeier et al [15] define MWL as the “proportion of the operator’s information processing capacity or resources that is actually required to meet system demands.” Gopher and Donchin [16] state that “mental workload may be viewed as the difference between capacities of the information processing system that are required for task performance to satisfy performance expectations and the capacity available at any given time.” They define MWL as a latent variable relating to the interaction between the operator and the task. As per Proctor [6], the definition of MWL is “a task [that] represents the level of attentional resources required to meet both objective and subjective performance criteria, which may be mediated by task demands, external support and past experience.”

**Figure 1:**
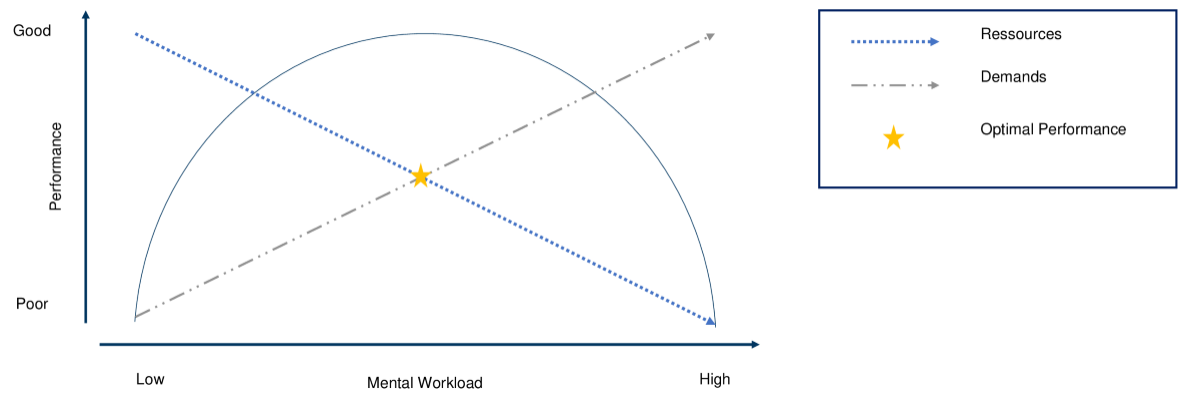
Task demands and limited resources result in different workloads and performance aspects. The optimal performance can be reached when resources and demands are balanced and the level of mental workload is moderate. Figure 1 is based on the representation of the Yerkes-Dodsen law [17].

In summary, there is no all-encompassing, universally accepted definition of MWL. We define MWL as a construct that addresses the influence of task demands on operator resources resulting in an impact on psychological factors such as performance (Figure 1) but not in the sense of stress or acceptance.

Especially during work, inadequate workload results in poorer performance [18]. Following the above definitions, a high workload can either be caused by unsuitable task requirements or by limited resources that are available in a cognitive manner, for example certain parts of the brain. The aim of measuring MWL is to determine the tasks and work processes that cause adverse or inappropriate levels of demands to draw conclusions about user performance as well as error prevention. Furthermore, the measurement of MWL can help identify factors that cause consequences such as technostress or burnout among nurses and physicians [10].

### Assessment of Mental Workload

MWL assessment was first developed and applied in other safety-critical environments such as aviation or aerospace or nuclear power plants. Owing to similar conditions—already described—in the sociotechnical system, workload assessment is also a useful approach in the clinical setting.

The assessment of MWL can be performed by different techniques. A distinction between analytical and empirical methods may be drawn. Analytical methods tend to be used in system development, while empirical methods are used when workload is to be measured directly in the executing system or in the simulation [13].

Analytical assessment methods are simulation models, expert opinions, or task analyses. Empirical methods are distinguished into three different categories: performance measures, subjective methods, and physiological techniques [6]. Performance measures refer to the measures of the primary and a secondary task.

Depending on the situation and the underlying question, one or more of these techniques are appropriate to apply. Several factors should be considered when selecting assessments, including sensitivity, diagnostic ability, intrusiveness, validity, reliability, simplicity of use, and user acceptance [19].

Tao et al [20] analyzed the physiological assessment of MWL across different application areas. One main result was that MWL assessments were not essentially valid in all areas, for example, for all tasks and differed in their validity.

Charles and Nixon [21] provide an overview of physiological measures that discriminate between different MWL levels. They detect varying ranges in the sensitivity of these measures but provide an evidence base for their deployment.

These reviews concentrate on physiological measures, not on all possible assessments. Although physiological measures are gaining relevance in the field of MWL assessment, methods that can be applied quickly and easily can still probably be helpful, especially in the health care sector.

### Objectives

The workload in health care institutions is high. A possible factor contributing to high workloads could be the use of DHT.

MWL is possibly the construct that can reflect best the workload caused by technologies. There is only light evidence for causes of MWL related to DHT. One reason might be that the health care sector has not been in the spotlight for researchers of human factors until now. To our knowledge, there currently is no review of the measurement methods for MWL caused by DHT.

As a primary objective, this systematic review intends to identify the impact of digital technologies, particularly HIS, on the workload of health care workers.

There are specific reviews investigating physiological methods assessing MWL as well as several papers studying the MWL in health care in general. We aimed to present a broader approach by looking at all methods that were used in the defined field while providing a more specific approach in focusing on DHT in particular, thus differing from already existing reviews to this topic [20,21]. We concentrated on a review of applied methods as well as their quality criteria. In addition (as secondary objectives), we aimed to assess what methods are being applied in health care to measure MWL relating to DHT. In particular, the application of eye tracking or pupillometry as a measurement method was investigated.

The research questions for this study are as follows:

1. In what manner do DHT contribute to the overall MWL of health care workers and which aspects or factors of DHT contribute to an increase in MWL?
2. What are the methods or assessments being applied to measure MWL related to HIS or digital technologies?
  What role does eye tracking or pupillometry play in context of measurement?
  What outcomes are being assessed via eye tracking?

### Rationale

Many different factors have led to a significant increase in workload in the health care sector in the past few years [22]. Work-related stress has become one of the main challenges in the health care sector [23]. Nurses in particular report high levels of work-related stress that lead to negative physical and psychological effects for them as well as for their patients [24]. Many nurses describe themselves as feeling empty and report depressive symptoms [25,26]. In Germany in particular, the number of days of sick leave taken by nurses is increasing every year. In addition to musculoskeletal diseases, which account for the majority of sick leaves, absences because of mental illness are increasing significantly [27]. The past two years (2020-2021) brought about many other challenges as well.

## Methods

### Study Registration

This systematic review is registered with PROSPERO (International Prospective Register of Systematic Reviews; CRD42021233271) and follows the PRISMA (Preferred Reporting Items for Systematic Reviews and Meta-Analyses) 2020 guidelines [28].

### Eligibility Criteria

We defined the inclusion criteria for this systematic review according to the population, intervention, comparison, outcome, context scheme and the corresponding research question or questions. The inclusion criteria related to the study population, measurement type (intervention or comparison), the outcome of the study, and the study setting (context). An additional inclusion criterion related to study design.

#### Study Design

This systematic review comprises 2 research questions. For both of these, we have included randomized controlled trials (RCTs), quasi-RCTs, case-control studies, and comparative cross-sectional studies as well as longitudinal design studies that either compare measurement methods for question 1 or generally measure MWL in the context of HISs and DHT.

#### Study Participants

We focused on HCPs who worked with DHTs that are directly related to patient care. These can be nurses, physicians, radiology assistants, medical students, or other clinicians. It is essential that the participants are supported by the HIS or DHT in their daily work with patients. We excluded studies that focused only on patients’ views on DHT use.

#### Intervention or Measurement

We included studies measuring MWL related to DHT that were directly related to patient care. The studies should have investigated whether there is a direct or indirect effect of DHT on workers’ MWL. Because the second research question evaluates the extent to which eye tracking is commonly used as a measurement method, we put a special focus on the inclusion of studies that apply eye tracking.

#### Study Setting

All types of study designs reporting original primary data as well as systematic reviews that adhered to our other inclusion criteria were included. We excluded commentaries, letters, and guidelines as well as scoping and narrative reviews.

#### Exclusion Criteria

We excluded studies that focused on the measurement of MWL in other contexts than health care (eg, aviation) as well as studies that were related to the measurement of allied constructs such as technostress or that focused on sources of MWL in health care other than DHT. In addition, we did not include studies that examined the workload of patients.

### Outcomes

The primary outcome of this systematic review was to analyze the influence of DHT on the MWL of HCPs and medical or nursing students.

Secondary outcomes included the types of assessments that are applied to measure MWL related to DHT. Additionally, we examined the impact of eye tracking on the measurement of MWL related to DHT.

### Information Sources

The following databases were systematically searched between January 20, 2021, and February 28, 2021, by using defined keywords (and synonyms) such as “mental workload,” “health information system,” “assessment,” “health care professionals” and “eye tracking” that result in specified search strings (the block chain is shown in Multimedia Appendix 1): MEDLINE (PubMed), Web of Science, Academic Search Premier and CINAHL (EBSCO), and PsycINFO. In addition, we searched for relevant research in the reference sections of included studies as well as of relevant recently published reviews. The keywords were defined by reviewing thesaurus systems such as Medical Subject Headings, expert opinions, and reviews of relevant studies.

We updated our search in February 2022 by replicating this process.

Following PRISMA, we organized the search terms by database and research question in a separate document [28]. We have attached this document (Multimedia Appendix 2)

### Search Strategy

The search strategy included the following four categories, each represented by keywords and synonyms: technologies used (eg, HIS), population (eg, HCPs), methods (eg, assessment), and MWL. In addition, eye tracking was added for research questions 2.1. and 2.2. The terms are linked by the Boolean operators AND or.

We restricted our search to articles published in the period between 2000 and 2022. This search time frame was chosen because it documents the development of the current generation of prehospital communication technology, such as telemedicine and electronic patient care reports [29]. The literature search was limited to articles written in English or German, as both reviewers were sufficiently proficient in these languages.

### Study Records

#### Data Management

Citavi (Citavi 6 for Windows–Campus; QRS International) was used for literature handling, that is, importing of articles and further screening of the literature. The *Rayaan* web-based screening tool was used to support further abstract screening and full-text analysis in a structured format [30]. In this context, the inclusion and exclusion criteria were also provided, functioning as the basis for the analysis process. The included articles were then imported to an extraction sheet.

#### Selection Process

**Figure 2:**
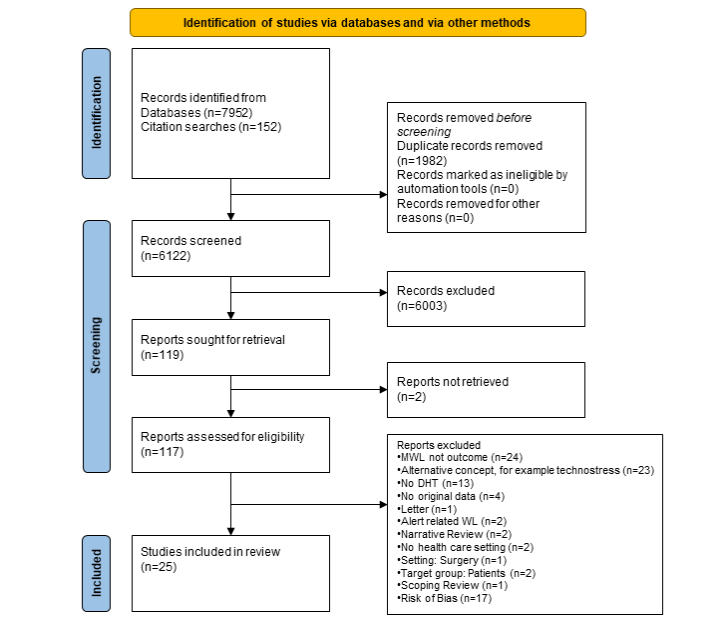
The figure displays the Flow Chart of the Search strategy starting with 8104 articles and resulting in 25 included studies. Most studies were excluded because MWL was not the primary outcome or the study focused alternative concepts [29]. DHT: digital health technology; MWL: mental workload.

The selection process was performed by two reviewers, LK and BB, (and two conciliating reviewers, ML and RR) according to PRISMA guidelines and is displayed using a flowchart (Figure 2) First, both reviewers assessed the studies regarding the inclusion and exclusion criteria for abstract screening. We included studies that (1) focused on DHTs such as HISs that are directly related to patient care, (2) focused on the MWL of HCPs that is related to DHT/HIS, (3) assessed MWL or cognitive load related to DHT, and (4) were processed in a health care context. We excluded studies that (1) focused on the assessment of MWL in other contexts (eg, aviation), (2) were related to the assessment of allied constructs such as technostress (3) that focused on patients (relating to either technologies or workload) (4) that focused on MWL not related to DHT and (5) were nonoriginal works (letters, guidelines, and narrative reviews) and books. In the next step, the full texts of the resulting studies were assessed independently.

Finally, we searched the references of the papers for further possibly eligible studies. In case of disagreements in any of the phases, a discussion between the two reviewers (LK and BB) based on the inclusion criteria was first attempted. If the discussion turned out to be inconclusive, a third reviewer (ML and RR) was involved.

#### Data Collection Process

A tabular extraction sheet for data extraction was used based on the outcomes of the review. To ensure uniformity across reviewers, we conducted a pretest standardization exercise before starting the data extraction process. Each reviewer extracted the themes of interest to an extraction sheet.

### Risk of Bias in Individual Studies

Two evaluators independently rated the quality of the identified studies using the QualSyst Scale [31]. Disagreements were resolved via discussion (among LK and BB) or, if necessary, resolved by a third reviewer (ML and RR).

Studies were rated using a structured tool (comprising 14 items). If a study completely fulfilled a criterion, it was assigned 2 points. In case of partial fulfillment, 1 point was assigned. If the criterion was not fulfilled by the study, no point was assigned to the study.

If a criterion was not applicable to the study presented (eg, blinding of the investigator), it was removed from the assessment. The achieved points as a percentage of the possible total points were evaluated as per the following criteria: a score of <0.5 by both reviewers resulted in exclusion, studies with scores between 0.5 and 0.65 were classified as having a moderate risk of bias, and studies with scores >0.65 were classified as having a low risk of bias.

### Data Items

LL and BB read the full texts and extracted information concerning identified and relevant aspects of the studies. We differentiated main study characteristics, measurements, and outcomes from relevant findings and recommendations.

In addition to the descriptive presentation of study characteristics and findings, we aimed to extract factors or aspects of DHT that contributed to an increase in MWL. Furthermore, we extracted information on how the included studies assessed the workload and in which settings eye tracking was used with regard to specific outcomes. On the basis of this, we developed an overview of the methods that can be used to measure MWL caused by DHTs meaningfully and validly. Furthermore, we assessed the studies concerning the categories of types of DHT and factors that contribute to a lower MWL.

The methods, settings, and outcomes were organized into logical categories that were rated by the reviewers. The typical categories of methods referring to MWL assessments were analytical or empirical techniques. Typical categories for settings were laboratory or field. Categories referring to assessed outcomes have to be defined during the reviewing process. In each category, we extracted how often an indicator for a category was applied (eg, category % = method applied/N studies) and how often combinations of specific indicators were used (eg, for total percentage with method A with setting B and outcome C, total % = combination applied/N studies). A typical indicator for category methods would be a questionnaire or subjective method. If an indicator was identified, the reviewers filled in the row with a 1; if no indicator was identified, for example, if the method was not applied, the table was filled in with a 0.

### Data Analysis and Synthesis

After the initial screening of the search results, we did not conduct a meta-analysis because the results and quantifications of the measures varied widely. Instead, we performed descriptive analysis to summarize the data, in which we first compared the studies in terms of the evaluation methods used (qualitative, quantitative, or mixed methods) and then performed a comparison of their survey methods.

We used the following two nonquantitative approaches for data synthesis: tabulation and a narrative approach.

In a first step, all main characteristics of each study were extracted (study design, the setting of the target population such as a hospital, sample size, age, sex, and population type such as physicians). We only included studies with a sample size of under 20 participants provided that the risk of bias was adequate [32].

We analyzed studies in terms of objectives, outcomes, and assessments as well as types of DHT. The quality criteria of assessments and information regarding the application of eye tracking as well as outcomes assessed via eye tracking were extracted. Differing from our protocol. we did not assess data on overall MWL in studies in addition to MWL levels related to DHT because the studies did not contain this information.

All included studies were evaluated with regard to their risk of bias.

A textual narrative synthesis of all included studies was made and comparable findings were synthesized. In addition, a descriptive analysis of eye tracking measures was extracted.

### Registration and Protocol

In the ongoing process, we had to perform a few amendments.

Contrary to what was defined in the protocol to this review, research questions 1.1 and 1.2 were not substituted to this final paper [32]. Deviating from the protocol’s attempt, we decided to use a different assessment tool to evaluate the risk of bias (QualSyst, [31]). In contrast to our protocol, we also included studies with a sample size under 20 participants under the condition that their risk of bias was adequate. Deviating from our protocol. we did not assess data on overall MWL in studies as well as MWL levels related to DHT because the studies did not contain this information.

## Results

### Search Strategy

**Figure 3:**
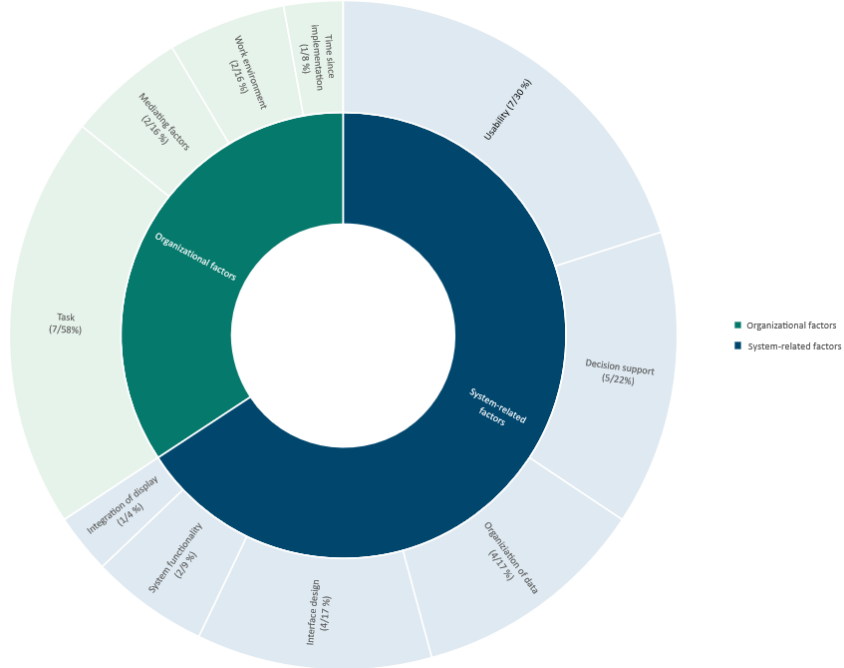
Contributing factors to mental workload related to digital health technologies grouped into system related and organizational factors. The categories are not disjunct, meaning that two categories may have been selected for one study. The categories are not mutually exclusive either.

The database search resulted in 7952 hits. Additional searches in the bibliographies of the identified publications and through discussions with experts yielded 152 more search results (N=8104). After removal of duplicates**,** 6122 (75.54%) publications remained in the review process. On the basis of the title and abstract screening, 6003 (74.07%) publications were excluded. Of the remaining 117 (1.4%) that were included in the full-text analysis, 72 (62%) were excluded for the following reasons: another concept of stress than defined in our paper (eg, technostress) was used, DHT was not part of the study, the study outcome was not workload, the paper was not an original work, the scope of the paper was alert-related workload, the population consisted of patients, it was a scoping or narrative review, there was no health care setting, or the full text was not available. In total, 46 (0.6%) studies were included in the qualitative synthesis and assessed for risk of bias. Of these, 17 (37%) studies were excluded because of their high risk of bias. The systematic search and the search strategy that followed resulted in 25 included studies (Figure 3).

### Risk of Bias Assessment

In total, 17 (37%) studies had a high risk of bias and were therefore excluded from the review because of scores <0.5.

A total 15 (33%) studies had scores between 0.5 and 0.65 and were therefore considered to have a moderate risk of bias. Furthermore, 10 (22%) studies had a low risk of bias (as shown in Multimedia Appendix 3; interrater agreement on scoring was *r*=0.91; *P*=.01).

Discrepancies in scoring generally resulted in different scores for item 1 (objectives) or 7 (blinding).

### Main Characteristics of the Included Studies

The main characteristics of the included studies are displayed in Table 1. Most studies were published between the years 2010 and 2022 [33-54]. Only 2 studies were published between the years 2002 and 2009 [55,56]. Most studies were conducted and published in the United States [33,35,37-40,42-50,52,53,55,57].

Most studies were carried out in laboratory or simulation settings [33,35,36,38-43,46-50,52,55,56], a few were done in field settings [37,45,54,57], and some were conducted only on the web [34,44,51].

A total of 10 studies were quasi-experimental [33,36,40,41,46,47,49,50,55,57], 8 were cross-sectional [34,37,39,44,48,51,54,56], 2 were observational [38,53], 1 was a longitudinal design study [45], and 4 were RCTs [35,42,43,52].

The included participants consisted of physicians (14 studies [33-35,37-39,42-44,46-52,54,56,57]), nurses (4 studies [40,45,53,55]), and medical or nursing students (1 study [41]) as well as mixed populations out of these 3 groups (6 studies [36,37,42,47,50,57]). The sample size in most included studies ranged from 10 to 50 participants [33,35,36,38-40,42,43,46-50,52-57], 1 study ranged from 50 to 100 participants [34], and 5 studies included >100 participants [37,41,44,45,51]. Furthermore, 16 studies reported times of experience with DHT [33,34,36,40,42,43,45,48,49,51-53,55-57].

**Table 1:** Main characteristics of the included studies, including the display of sample statistics, setting, study design, and descriptive information about the included studies.

Author	Country	Setting	Study design	Sample size, n	Age (years), mean (SD) or median (IQR)	Sex, n (%)	Experience with DHT^a^ (years)	Occupation
Ahmed et al [33], 2011	United States	L^b^	QS^c^	20	NR^d^	NR	>1 year	P^e^
Ariza et al [34], 2015	United Kingdom	Wb^f^	CS^g^	67	NR	NR	6.7 years	P
Carayon et al [35], 2020	United States	L	E^h^	32	NR	Female 8 (25); male 24 (75)	NR	P
Currie et al [36], 2017	United Kingdom	L	QS	S^i^ 37; N^j^ 11	S 27.31;N 31.91 SD or range NR	NR	S 0 years; N 8.73 years	N; S
Dunn Lopez et al [57], 2021	United States	F^k^	QS	N 22; *P* 13	N 32.5 (20-66); *P* 45.3 (25-63)	N: female 19.8 (90), male 2.2 (10); P: female 5.98 (46), male 7.02 (54)	N 2.5 years; P 6.2 years	N; P
Grünloh et al [54], 2016	Sweden	F	CS	12	NR	Female 5 (42); male 7 (58)	14 (2-30) years	P
Holden et al [37], 2015	United States	F	CS	170	NR	Female ≥161 (>95) Males: <9 (<5%)	NR	N; P
Khairat et al [38], 2018	United States	L	O^l^	14	Resident: 18-34 years (6, 100%) Attending: 35-50 years (7, 87.5%) 51-69 years (1, 12.5%)	Female 7 (50); male 7 (48)	Residents 3 years; Attending >3 years	P
Khairat et al [39], 2019	United States	L	CS	25	33.2 (6.1) years	Female 13 (52); male 12 (48)	NR	P
Koch et al [40], 2012	United States	L	QS	12	31.5 (23-57)	Female 8 (66); male 4 (34)	Self-rated experts 9 years; self-rated novices 1 year	N
Lyell et al [41], 2018	Australia	L	QS	120	24.5 (2.99)	Female 55.2 (46.7); male 63.6 (53.3)	NR	S
Mazur et al [42], 2015	United States	L	E	29	NR	NR	WebCIS 0.5-3 years; Epic 0.5 years	P; S
Mazur et al [43], 2019	United States	L	E	38	NR	Female 25 (66); male 13 (34)	Residents 36 years; fellows 2 years	P
Melnick et al [44], 2020	United States	Wb	CS	848	53 (28-84)	Female 509 (58.1); male 353 (40.6)	NR	P
Moreland et al [45], 2012	United States	F	Lo^m^	719	38.5 (11.2)	Female 650 (90.9); male 69 (9.1)	Participants self-rated “comfort with system” and sorted by group (n) Novice 41; knowledge of basics 288; experts 390	N
Mosaly et al [47], 2018	United States	L	QS	17	NR	NR	NR	P
Mosaly et al [46], 2019	United States	L	QS	38	NR	Female n (63); male n (27)	NR	P; S
Pollack et al [48], 2020	United States	L	CS	29	43 (35-58)	Female n (48); male n (52)	11 (3-30) years	P
Richardson et al [49], 2019	United States	L	QS	32	39.29 (12.4)	Female n (50); male n (50)	Participants (n); Residents (minimum of 3 years experiece) 16; attending physicians (Training level) 16	P
Saleem et al [55], 2007	United States	L	QS	16	NR	NR	None	N
Sampson et al [50], 2019	United States	L	QS	35	34.2 (25-59)	NR	NR	N; P
Shachak et al [56], 2009	Israel	L	CS	25	NR	Female n (56), male n (44)	6.8 years	P
Shah et al [51], 2016	United Kingdom	Wb	CS	188	NR	Female n (63.3); male n (36.7)	3-6 months: 54 (n) 6 months – 1 year: 51 (n) >1 year: 83 (n)	P
Wanderer et al [52], 2011	United States	L	E	20	NR	NR	Residents 10 years; attending physicians 10 years	P
Yen et al [53], 2020	United States	F	O	7	30 (6)	Female 6 (86); male 1 (14)	NR	N

^a^DHT: digital health technology.

^b^L: labor.

^c^QS: quasi-experimental.

^d^NR: not reported.

^e^P: physician.

^f^Wb: web-based.

^g^CS: cross-sectional.

^h^E: experimental.

^i^S: student.

^j^N: nurse.

^k^F: field.

^l^O: observational.

^m^Lo: longitudinal.

The included studies did not apply a homogenous definition approach for MWL: 13 studies did not provide a definition of their underlying concept at all [35,36,38,40-43,45,50,52-54,57], 2 studies applied a classic definition of MWL [37,51], 3 studies defined MWL as mental effort [34,46,47], 2 as information overload [33,39], and 5 studies applied a definition of cognitive load [41,44,48,49,56]. All the applied definition had a common base that could be summed up under the concept of MWL that we defined for inclusion.

The analyzed types of DHT were grouped into one of six categories as appropriate electronic health records or electronic medical records (EMRs), computerized decision support systems, information display or vital sign display, e-prescribing systems, anesthesia system, and computerized clinical reminders. More than half of the studies (13/25, 52%) analyzed electronic health records or EMRs.

### Research Question 1: Contribution of DHT to the MWL of HCPs

Studies with various outcomes reflecting the association of DHT and MWL were included.

Overall, 20 (83%) of the included studies investigated the MWL related to DHT in general [33,38,40,53,54,56], 8.33% (2/25) compared MWL before and after redesign of DHT [51,52], and 12.5% (3/25) of the studies analyzed MWL before and after implementation of a new DHT [37,50,55]. A further 12.5% (3/25) of the studies compared MWL among different DHT or systems [34,35,40].

Furthermore, 33^,^33% (5/25) of the included studies investigated the relationship between the usability of the DHT and MWL [39,43,44,48,57], 16.67% (4/25) assessed MWL related to task demands and performance during the use of DHT [39,42,46,47], 8.33% (2/25) of the studies examined the influence of decision support on MWL [41,49], and 4% (1/25) examined other influences [36].

The included studies identified various factors of the systems that contributed to the MWL of HCPs. Some factors were rooted in the systems themselves; other factors were caused by influences and circumstances on an organizational level. We grouped the results by organizational and system-related factors (Figure 2).

### Organizational Factors

A total of 8 studies identified the task to be performed by the use of the DHT as the relevant factor that contributes to an increasing MWL [34,36,37,41,42,47,50,54,56]. In all cases, the tasks did not fit the processes already implemented in the system.

Of these, 2 studies stated the overall workload in the working environment as the contributing factor [53,54]: the higher the general workload, the higher the MWL related to DHT.

Other relevant organizational factors that were identified by a study was the amount of time since implementation [45]: the longer a system was implemented, the lower the MWL, which initially increased significantly immediately after implementation.

In addition to direct influences, a study examined mediating factors and specifically identified gender and total hours worked. Women, as well as those who worked fewer hours, had a smaller increase in MWL from the DHT [44,57].

### System Factors

In addition to organizational factors, most studies (23/25, 92%) identified factors based predominantly in the underlying system of the DHT.

A total of 4 studies cited weaknesses in the interface design as main factors for an increasing MWL of HCPs [39,48,50,51].

In addition to the interface design, 6 studies identified deficiencies in the usability as an influencing factor for increasing workload [39,43-45,50,51]. Studies refer to longer task completion times, higher error rates, a higher number of clicks, and differences in usability ratings between men and women (women contributed to higher rankings) [39]. There were also reports of less MWL because of automatically sorted and displayed test results in an electronic health record [43] and a significant correlation between MWL and usability [44].

A further 5 studies identified nonfunctioning decision support as a critical factor in increasing MWL [35,41,47,49,56], and 4 studies detected the organization of data and information as influencing factors [33,36,38,56].

A study showed that integrated displays cause less MWL than nonintegrated traditional displays [40]. In addition to precisely identifiable factors, 2 studies indicated that high MWL is particularly because of system functionality of the system in itself [34,39].

### Research Question 2: Assessment Methods of MWL Related to DHT in Health Care

#### Overview

**Figure 4:**
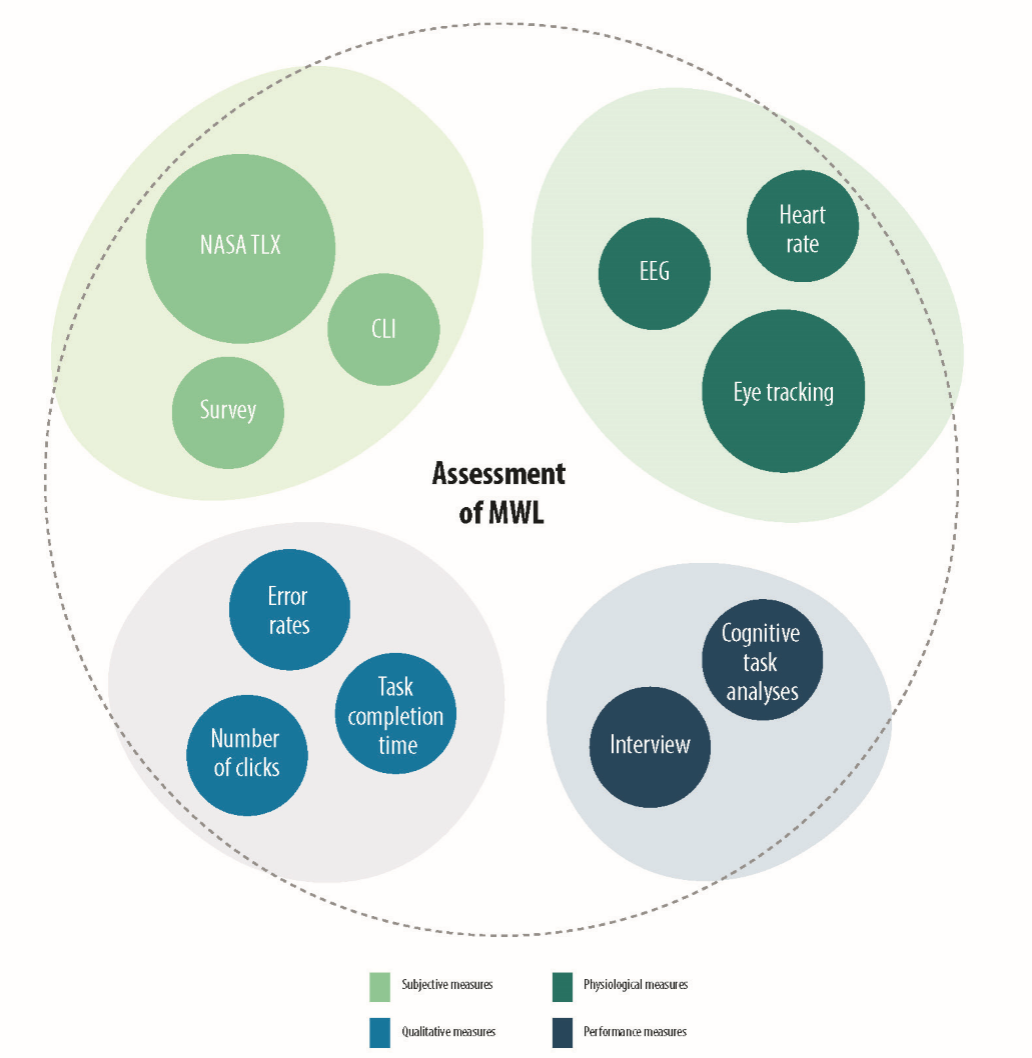
Identified assessment methods grouped by assessment type. Most applied assessment type were subjective methods – NASA TLX was the assessment that was used in most studies. The size of the circles is proportional to the frequency of application in the studies.

All applied and identified assessment methods have been empirical. A total of 18 studies applied subjective methods [34-36,38,40-42,44,48-50,52,53,55,57], and 2 studies used performance measures [33,35]. Furthermore 5 studies used physiological methods [36,42,43,46,47], and all of them applied eye tracking techniques—either isolated or in combination with other measures [36,42,43,46,47]. In a study, an interview was conducted [54], and a study used the cognitive task analyses technique [56]. The identified measures are displayed in Figure 4.

#### Subjective Measures

##### National Aeronautics and Space Administration–Task Load Index or Raw–Task Load Index

A total of 52% (13/25) of the included studies applied the National Aeronautics and Space Administration (NASA)–Task Load Index (TLX) or an adapted form of the questionnaire such as the Raw-TLX to assess the MWL of HCPs in relation to DHT [34-36,38,40,42,44,48-50,52,53,55,57].

Of these, 3 (12%) studies adapted the NASA-TLX in form of the Raw-TLX based on numerous trials [34,35,51].

The NASA-TLX is a very commonly applied subjective assessment method to assess the MWL related to a specific task. The NASA-TLX has been applied mostly for questions of interface design and evaluation [58] and is often combined with other applied measures such as performance measures [58].

The questionnaire consists of six scales that each represent 1 dimension: MWL, physical workload, temporal workload, effort, frustration, and performance [59].

The original form of the NASA-TLX provides a rating scale ranging from 0 to 100 and a weighting of the different values of the scales [59]. However, several studies could show that the weighting of the scales in particular has no degrading influence on the sensitivity of the scales [58]. Thus, this form of the questionnaire is called the Raw-TLX and is the most commonly used version along with the NASA-TLX itself [58]. Even a change in the Likert scale does not seem to lead to a strong modification of the sensitivity or the quality criteria [58]. The psychometrics for both versions, the original NASA-TLX and the Raw-TLX, can be considered good [60,61].

##### Cognitive Load Inventory

The cognitive load inventory was applied by a study and can be defined as a subjective cognitive load measurement tool [62]. Leppink et al [62] developed a 10-item questionnaire, rated on a 10-point Likert scale with the dimensions of intrinsic, extraneous, and germane load. The development of this scale was based on the cognitive load theory [63]. Previous research shows that psychometrics for this scale can be considered good [62].

##### Self-developed Surveys

A study used a self-developed survey that consisted of items for external (3 items) and internal (2 items) MWL. The Cronbach α for both scales was average to good [37].

Another study analyzed nurse workload by using 2 self-developed items that were rated on a 10-point Likert scale. Content validity (0.92) and internal consistency can be considered good (Cronbach α=.89-.95) [45].

#### Physiological Measures

##### Electroencephalography

Mazur et al [42] measured cognitive workload derived from electroencephalography. They processed the data by applying the ABM’s algorithm that automatically calculates the index of cognitive workload.

Previous research shows that specific features of brain activity are good indicators for MWL; for example, theta activity increases with increasing mental effort [64].

Accuracy levels of electroencephalography measures can be classified as average (approximately 60%) [65].

##### Eye Tracking

A total of 5 studies applied eye tracking to measure the MWL related to DHT (displayed in Table 2). Furthermore, 3 studies assessed the blink rate of participants as an indicator for MWL [43,46,47], 2 studies detected pupil dilations [42,46], 1 study assessed fixation frequency and visit frequency [36], and 1 study applied the measure of task evoked pupillary response [47] None of these studies reported quality criteria for their assessment.

**Table 2:** Display of assessments of mental workload (MWL) via eye tracking.^a^

Study	Measures	Measure combination	Outcomes assessed
Currie et al [36], 2018	Visit frequency; fixation frequency	Questionnaire (NASA-TLX^b^)	Automatic prediction of performance of nurses and interpretation of vital monitors
Mazur et al [42], 2016	Pupil dilations	Questionnaire (NASA-TLX); electroencephalography	Performance (error count and task completion time)
Mazur et al [43], 2019	Blink rate	N/A^c^	Mental and physical workload, performance, and fatigue
Mosaly et al [46], 2019	Blink rate; pupil dilations	N/A	Mental effort and performance
Mosaly et al [47], 2018	Blink rate; task evoked pupillary response	N/A	Mental effort and performance

^a^The most frequently applied measure was pupil dilation. The outcomes assessed varied across studies.

^b^TLX: Task Load Index.

^c^N/A: Not applicable.

##### Heart Rate

A study used a wearable heart rate monitor to detect heart rate changes as indicators of nurses’ workload levels. The device assessed biometric signals continuously with time stamps. No psychometric values were given [53].

#### Performance Measures

A total of 2 studies applied performance measures as detection methods for MWL. Both studies did not apply these as stand-alone assessments; they combined the assessments with questionnaires. The response time, error rate, and number of clicks were measured.

Ahmed et al [33] registered the time to task completion (in seconds). Completion of tasks on a standard EMR in comparison to a redeveloped one took twice as long.

Ahmed et al [33] also counted the number of errors. They identified 4 times as many errors per participant when using the standard EMR than when using a redesigned user interface.

Carayon et al [35] assessed the number of clicks and task completion time and correlated these using the measures of NASA-TLX. Physicians were faster and interacted with lesser interface elements for a clinical decision support system when compared with the standard system.

#### Qualitative Measures

Shachak et al [56] applied a cognitive task analyses using semistructured interviews as well as field observations to assess MWL related to EMRs. The interview is adapted from the study by Militello and Hutton and asks for characteristics of the system that require difficult cognitive skills, errors, and special attention. Physicians reported a reduced MWL when EMR systems were used

#### Quality Criteria of Applied Methods

Overall, 68% (17/25) of the included studies did not report any quality criteria or measure. Some referred to reliability scores cited from previous research.

Furthermore, 5 (20%) studies reported measures of reliability (Cronbach α). Carayon et al [35], Lyell et al [41], and Moreland et al [45] reported a Cronbach α between.8 and .9.

Holden et al [37] and Shah and Peikari [51] reported a Cronbach α between.7 and .8.

In addition to Cronbach α, Moreland et al [45] reported a high content validity (0.9). A total of 2 (8%) studies reported quality criteria but only partly or not adequately [40,57].

### Approach Toward the Most Applied Combination or Gold Standard

The combination of setting and applied measure that was detected in most cases was a laboratory setting combined with a subjective measurement method. Further, it can be identified that the outcome relationship between MWL and usability related to DHT measured by subjective methods or performance measures in the laboratory was established in most cases. Other frequently applied combinations were subjective method, MWL related to DHT, decision support, usability, system comparison, or other as well as physiological measures combined with task demands or other, in the laboratory. The results of the combinations of settings, assessments, and outcomes are displayed in Multimedia Appendix 4.

## Discussion

Although several measures are applied frequently in the assessment of MWL in varied areas, the use of these methods may be limited by shortcomings in terms of knowledge about their correct and valid application in the field of human-technology interaction in health care. Therefore, our review had 2 separate but related objectives as described in the following sections.

### Principal Findings

This systematic review investigated 25 studies that applied various measurement methods to assess the MWL related to DHT. The aim of the review was to show which factors of DHT contribute to a high MWL for HCPs in health care settings. In addition, the review was intended to identify methods that are currently used to measure MWL in health care. In this context, the role of eye tracking as a measurement method in particular was considered.

The following aspects can be considered the most relevant while summarizing the main results:

- First, the investigation showed that self-report subjective measurement methods (eg, the NASA-TLX), are the most frequently applied measures and can be considered the most prominent measure in MWL evaluation. Studies are most commonly conducted in laboratory settings. If physiological measures such as eye tracking are applied, they are combined with other measurement methods.
- Although a most frequent approach could be identified, it has to be stated that the methods used for the measurement of MWL related to DHT varied in their scope, methodology, outcomes, and evidence level as well as results concerning the MWL created by DHT.
- The risk of bias assessment revealed severe deficiencies in most studies because of methodological issues, inadequate sample sizes and statistical power, and poor study designs as well as deficient conduction of studies.

In particular, the negative effect of DHT on MWL in health care was consistent across studies. At the same time, DHT could support HCPs, but it must fulfill different criteria to achieve this. In addition to the system-related factors, organizational issues contribute to the influence of DHT on high MWL.

### Comparison With Prior Work

Consistent with previous reviews, we identified the application of subjective measurement methods to be the most frequently used approach for the assessment of the MWL. [66]. Although we were able to identify a most frequently applied method, one of the main findings of this review was the heterogeneity of applied assessments, which is also in line with previous analyses [20,66]. Some studies used a combination of methods; for example, eye tracking and NASA-TLX. Reviews that investigate methods to measure MWL usually focus on 1 type of method, such as physiological measures [17,18], or a specific field of application (eg, driving distraction) [62]. The health care domain—although it can be seen as a safety-critical environment—was not the focus of these reviews. Charles and Nixon [21] included 58 studies in their review, none of which addressed MWL in health care. First, while other studies focused on nonhealth care domains, our review revealed methodological shortcomings in the health care area.

Second, our idea was to provide a holistic review of methods being used for the application of DHT in health care.

Previous reviews also checked for combined measure assessments; in line with our findings, Charles and Nixon [21] and Tao et al [20] found several studies that combined physiological measures and the NASA-TLX.

In contrast with our findings, Charles and Nixon [21] found many studies reporting quality criteria such as sensitivity and validity, also for physiological measures. However, they found differences for validity and sensitivity of measures comparing field and laboratory settings. This finding corresponds to the findings of Tao et al [20] and also partially to our findings.

Kabilmiharbi et al [22] reviewed studies concerning multiple driving distractions. In contrast to health care settings, MWL assessment during driving is mainly conducted via physiological or performance measures [63]. In line with our results, NASA-TLX was the most commonly used subjective assessment.

We identified 4 different eye tracking measures applied in the studies included in our review (fixation frequency, blink rate, pupil dilation, and visit frequency). Tao et al [20] identified blink rate, pupil diameter, and fixation duration as correlates of MWL, but—in contrast with our results—identified additional eye tracking measures that were relevant.

Besides a strong heterogeneity, a rather homogeneous approach with regard to the setting was revealed. This is equivalent to findings of Tao et al [20]. Most studies were performed in the laboratory. Outcomes differed marginally but were still differentiated for more discriminative analysis.

Factors contributing to MWL in health care can be identified as occupational or individual. Occupational factors can be level of education, type of working unit (eg, intensive care unit), work shifts, and number of patients under care [64]. Studies from other domains show, for example, an enhancement in situation complexity, task-related and individual factors as well as organizational factors such as time pressure as possible predictors of MWL [65]. However, none of the studies mentioned in this section explicitly addresses the relationship between MWL and HIS/DHT.

Many studies also consider MWL as a starting point for further consequences on the performance of the HCPs, for example, a hazard to patient safety or job satisfaction (66), rather than the factors contributing to a high MWL.

### Strengths and Limitations

This review has some limitations with respect to the included studies.

First, because of the heterogeneity of the assessment methods, analyses, and study designs of the included studies as well as their methodological quality, a meta-analysis could not be conducted.

Second, many studies performed retrospective measurements of MWL that did not allow for causal conclusions in the results. The restriction of causality is further limited by nonreported quality criteria.

Third, the results as well as the review itself are further limited by the search process. Part of the results are aspects of factors that contribute to MWL related to DHT. These aspects were not explicitly searched for in the literature examination. It can therefore be assumed that not all relevant studies concerning these factors have been included. The search process can also be considered to be limited in the sense that it became apparent during the review process that many authors integrate the constructs of mental or cognitive workload into other constructs or refer to concepts similar to these. Other constructs that may follow a similar definition, such as mental effort, were not considered in this search. It can therefore be assumed that these studies were not included in the review.

The definition of the MWL construct was not consistent across the studies examined. In addition to MWL, stress, cognitive load, fatigue, and mental effort, and other similar concepts have been grouped under the term information overload and limited workload capacity resulting from perceptual load. However, other studies have developed their own concepts (eg, stress related to information systems) that mean slightly different things but include parts of the definition of MWL. Our results are limited in terms of not including these studies as they also included aspects of stress (eg, acceptance) that do not refer to the MWL classification that was relevant for our paper.

However, in order to develop a gold standard for measuring MWL in health care settings, it seems highly relevant to precisely define the construct. Identifying studies referring to a selective definition of MWL was therefore particularly challenging for this review. Because of the strong heterogeneity of the research field, we cannot eliminate the possibility that some studies were not included, which were not identified by our search terms because of variations in construct naming.

The combination of the different approaches toward the assessment of MWL also showed strong heterogeneity. Some of the methods—especially the physiological ones—require extensive preparation and equipment and are very time-consuming, particularly in their evaluation. Thus, not every method can be considered suitable for every setting (eg, in a clinical setting).

The approach of analysis in the laboratory seems understandable on the one hand, because content validity and reliability are easy to achieve. On the other hand, the small number of field studies ensures that results cannot be transferred to other settings easily (external validity) and that various bias effects at least partly due to presumably weak quality of the study implementation also led to erroneous results. This also applies to the generalizability across populations; therefore, studies referring to MWL of patients were not included.

The applied quality criteria assessment revealed shortcomings in methodological quality across many studies. There was only a small amount of studies with a quality rating of >65% (10 studies [37,39,41-44,49,50,54-56]). However, a possible explanation for such a low rate might be that many of the remaining studies could be regarded as first or exploratory approaches.

Most studies did not report quality criteria such as content validity or reliability. Reliability indicates the degree to which an assessment can differ between high and low workloads [67]. Content validity refers to the degree to which an assessment reflects all aspects of MWL [67]. Studies that reported reliability measures reported acceptable to high levels of internal consistency of the assessments. Studies that reported content validity reported moderate levels of internal consistency of the assessments. To develop a gold standard in the assessment of MWL in health care, the reporting of quality criteria as indications for the quality of a measurement method is essential.

Studies that were not published in full text or in English were excluded; consequently, additional information on measurement properties and descriptions of methods for assessing masticatory performance that may have potentially affected the level of evidence might have been missed.

All included papers were published in the period between 2002 and 2022; the literature search was limited to papers with publication years between 2000 and 2022.

We detected an increase in the 2010s that could give a hint regarding the increasing interest in the topic during this time. On the other hand, the term MWL, as already described, was not defined in as much detail as it should have been. Therefore, the detected increase could have also been produced by more specific definitions in the last years.

In addition, it is possible that we did not find all relevant articles, despite having thoroughly defined which terms to include and having conducted a systematic search using Medical Subject Heading terms

### Future Directions

Our results show a very heterogenic approach toward the assessment of MWL related to DHT in health care settings. Although the assessments are heterogeneous, it can be assumed that there are 2 groups of contributing factors to MWL related to DHT, factors rooted in the system itself and organizational factors such as the task for which the system is being used.

When it comes to implementing or applying already implemented DHT in health care, these factors should be considered holistically.

The following steps should be taken for implementing and developing a gold standard and conducting future research in this field of study:

1. Conducting well-developed studies that take into account quality criteria and adequate sample sizes as well as effect size and power calculation. Future research is warranted to include HCPs with more diverse backgrounds (eg, differentiated by previous experience with DHT) and to have adequate statistical power for testing.
2. Reviewing MWL studies in related fields, such as power plants or aviation research.
3. Identifying methods that apply most to the research question being posed (eg, what is the amount of MWL of an intensive care unit nurse during a shift when switching between the EMR system and vital signs monitors), which would probably lead to a dynamic approach assessed by a dynamic assessment method such as eye tracking.

Future research is required to further investigate the relations between factors that might be contributing to MWL while using a DHT and MWL in general. Our results show a first step forward for grouping these factors. However, further primary research and review work is necessary for the development of a theoretical framework.

### Conclusions

Our review of 25 papers shows a diverse assessment approach toward the MWL of HCPs related to DHT as well as 2 groups of relevant contributing factors to MWL. The most frequently applied method has been the NASA-TLX (subjective measurement approach) in laboratory settings. The contributing factors can be divided into system-related factors and organizational factors.

Our results show a few new approaches being used for assessing MWL in relation to systems in a valid, reliable and practical way; eye tracking could be one of these measurement techniques.

Although methodological biases were identified, we recommend further research concentrating on adequate assessments of MWL of HCPs for relevant settings. We would also like to recommend the evaluation of quality criteria.

## Appendix

Multimedia Appendix 1 Block chain of keywords that were used to create the search terms.

Multimedia Appendix 2 Search string and results.

Multimedia Appendix 3 Display of assessment scores of Qualsyst tool of both reviewers (rater LK and BB).

Multimedia Appendix 4 Tabular display of the descriptive results relating to a combination of specific outcomes of the review: The table displays results of single categories (e.g. subjective method) on the one hand, and on the other hand combined results of several categories (e.g. applied method and outcome of study).

## Abbreviations

DHT: digital health technology
EMR: electronic medical record
HCP: health care professional
HIS: health information system
MWL: mental workload
NASA: National Aeronautics and Space Administration
PRISMA: Preferred Reporting Items for Systematic Reviews and Meta-Analyses
PROSPERO: International Prospective Register of Systematic Reviews
RCT: randomized controlled trial
TLX: Task Load Index
