# Supplementary material for: Examining Mental Workload Relating to Digital Health Technologies in Health Care: Systematic Review
Source: J Med Internet Res. 2022 Oct 28;24(10):e40946. doi: 10.2196/40946 (PMC9652730; doi:10.2196/40946)
Supplement: Multimedia Appendix 1 [file jmir_v24i10e40946_app1.doc]

|  | **Block 1** | **Block 2** | **Block 3** | **Block 4** | **Block 5** |
| --- | --- | --- | --- | --- | --- |
| ***Synonyms*** *(close search terms)* | Medical Informatics Applications | Eye Movement Measurements | Workload | Health Personnel | Technology Assessment |
| ***Related terms*** | Electronic Health Record(s), Health Information System(s), Health Technology | Eye tracking, saccade, fixation, eye gaze, eye tracker, | Employees’ workload, cognitive load, mental workload, Human Channel Capacity, Information Overload, Performance | Medical Personnel, Nurses, Physicians, doctors, laboratory assisstants, midwife | Health Information Systems evaluation |
| ***General term*** *(broader term)* | Medical Informatics | Physiological Measures | Human factors | Personnel | Psychometrics |
