# Supplementary material for: Examining Mental Workload Relating to Digital Health Technologies in Health Care: Systematic Review
Source: J Med Internet Res. 2022 Oct 28;24(10):e40946. doi: 10.2196/40946 (PMC9652730; doi:10.2196/40946)
Supplement: Multimedia Appendix 2 [file jmir_v24i10e40946_app2.pdf]

Search Strategy Documentation

Research Question	This systematic review aims to examine the Mental Workload of Health Care Workers that is related to Health Information Systems and Digital Technologies used in Health Care Settings.<br><br>Additionally, it intends to identify which methods/ assessments are used to measure Mental Workload relating to those Technologies in Health Care. In this context, we focus on the analysis of Eyetracking as measurement method.<br><br>a) What is the impact of Health Information Systems/ Digital Technologies used in Health Care settings on the Mental Workload of Health Care Workers?<br><br>b) Which methods/ assessments are applied to measure Mental Workload related to Health Information Systems/ Digital Technologies?<br><br>b.1.) Which role does Eyetracking/ Pupillometrie play?				
Information Sources	MEDLINE (PubMed), PsycINFO, Web of Science, EBSCO (Academic Search Premier & CINAHL)				
Source:		Search strategy used (keywords & Boolean)			

	Date of search		Search Limits or filters (e.g. dates, language)	# results found	Comments
Pubmed (Medline); Search 1	25.01.2021	((((((applications, medical informatics[MeSH Terms]) OR (record, electronic health[MeSH Terms])) OR (record, electronic medical[MeSH Terms])) OR (health information technology[MeSH Terms])) OR (design, medical device[MeSH Terms])) AND (((((employee workload[MeSH Terms]) OR (stress, psychological[MeSH Terms])) OR ("mental workload")) OR ("cognitive load"))) AND (((((health personnel[MeSH Terms]) OR (administrator, nurse[MeSH Terms])) OR (aide, nurses[MeSH Terms])) OR (assistant, physicians[MeSH Terms])) OR (physicians[MeSH Terms]))	2000-2021<br><br>English or German	373	20
Pubmed (Medline); Search 2	25.01.2021	((((((applications, medical informatics[MeSH Terms]) OR (record, electronic health[MeSH Terms])) OR (record, electronic medical[MeSH Terms])) OR (health information technology[MeSH Terms])) OR (design, medical device[MeSH Terms])) AND (((((employee workload[MeSH Terms]) OR (stress, psychological[MeSH Terms])) OR ("mental workload")) OR ("cognitive load"))) AND ((((((assessement) OR (questionnaire)) OR (survey)) OR (scale)) OR (test))	2000-2021<br><br>English or German	1209	10
Pubmed (Medline) Search 3	25.01.2021	((((((employee workload[MeSH Terms]) OR (stress, psychological[MeSH Terms])) OR ("mental workload")) OR ("cognitive load")) AND (((((eye movement[MeSH Terms]) OR (pupillometry)) OR ("eye tracking")) OR ("eye tracker")) OR ("eye movement measurement")) AND (english[Filter] OR german[Filter])) AND (Health Care)	2000-2021<br><br>English or German	21	6

EBSCO (Academic Search Premier / CINAHL) Search 1	25.01.2021	((electronic+health+records)+OR+(electronic+medical+record)+OR+(health+information+systems)+OR+(digital+health+technology)+OR+(electronic+health+record+system))+AND+((workload)+OR+(mental+workload)+OR+(cognitive+workload)+OR+(cognitive+load)+OR+(information+overload+AND+stress))+AND+((health+personnel)+OR+(health+care+workers)+OR+(health+care+professionals)+OR+(doctors+OR+physicians)+OR+(nurse))	2000-2021 English or German	633	141
EBSCO (Academic Search Premier / CINAHL) Search 2	25.01.2021	((electronic+health+records)+OR+(electronic+medical+record)+OR+(health+information+systems)+OR+(digital+health+technology)+OR+(electronic+health+record+system))+AND+((workload)+OR+(mental+workload)+OR+(cognitive+workload)+OR+(cognitive+load)+OR+(information+overload+AND+stress))+AND+((health+personnel)+OR+(health+care+workers)+OR+(health+care+professionals)+OR+(doctors+OR+physicians)+OR+(nurse))+AND+((measurement+tool)+OR+(scale)+OR+(test)+OR+(assessment)+OR+(questionnaire+OR+survey)+OR+(methods))	2000-2021 English or German	460	107
EBSCO (Academic Search	25.01.2021	((workload)+OR+(mental+workload)+OR+(cognitive+workload)+OR+(cognitive+load)+OR+(information+overload+AND+stress))+AND+((health+personnel)+OR+(health+care+workers)+OR+(health+care+professionals)+OR+(doctors+OR+physicians)+OR+(nurse))+AND+((eye+tracking+OR+eye+tracker+OR+eye+movement+measurements+OR+visual+tracking)+OR+(fixation)+AND+(pupillometry))	2000-2021 English or German	16	5

Premier / CINAHL) Search 3					
Web of Science Search 1	25.01.2021	TS=(Health Record* OR Electronic Health Record* OR Health Information System* OR Health Technology*) AND TS=(cognitive load OR workload OR mental workload OR information overload OR stress OR Human Channel capacity) AND TS=(cognitive load OR workload OR mental workload OR information overload OR stress OR Human Channel capacity)	2000-2021 English or German	1971	251
Web of Science Search 2	25.01.2021	TS=(Health Record* OR Electronic Health Record* OR Health Information System* OR Health Technology*) AND TS=(cognitive load OR workload OR mental workload OR information overload OR stress OR Human Channel capacity) AND TS=(cognitive load OR workload OR mental workload OR information overload OR stress OR Human Channel capacity) AND (TS=(measurement method* OR measurement OR assessment OR test OR scale OR questionnaire OR survey OR instrument*) NOT TS=(Screening) NOT TS=(diagnosis))	2000-2021 English or German	845	112
Web of Science Search 3	25.01.2021	TS=(Health Record* OR Electronic Health Record* OR Health Information System* OR Health Technology*) TS=(cognitive load OR workload OR mental workload OR information overload OR stress OR Human Channel capacity)		115	2

		TS=(eye movement* OR eye-tracking* OR saccade* OR fixation* OR psychophysiology OR eyemovements OR eyetracking)			
PsycInfo	10.01.2021	(“Health Record*” OR “Electronic Health Record*” OR “Health Information System*” OR “Health Technology*”)<br><br>AND<br><br>(“cognitive load” OR workload OR “mental workload” OR “information overload” OR “Human Channel Capacity”)<br><br>AND<br><br>(“health personnel” OR physician* OR nurse* OR doctor*)<br><br>OR<br><br>(“Health Record*” OR “Electronic Health Record*” OR “Health Information System*” OR “Health Technology*”)<br><br>AND<br><br>(“cognitive load” OR workload OR “mental workload” OR “information overload” OR “Human Channel Capacity”)<br><br>AND<br><br>(eyemovement* OR “eye-tracking*” OR saccade* OR fixation* OR eyetracking OR saccade* OR fixation*)	2000-2021<br><br>English or German	1882	

654 nach aktualisierter Suche
